# Research on Path Planning Algorithm of Driverless Ferry Vehicles Combining Improved A* and DWA

**DOI:** 10.3390/s24134041

**Published:** 2024-06-21

**Authors:** Zhaohong Wang, Gang Li

**Affiliations:** School of Automobile and Traffic Engineering, Liaoning University of Technology, Jinzhou 121001, China; 221285047@stu.lnut.edu.cn

**Keywords:** path planning, improved A* algorithm, improved DWA, fuzzy control, fusion algorithm

## Abstract

In view of the fact that the global planning algorithm cannot avoid unknown dynamic and static obstacles and the local planning algorithm easily falls into local optimization in large-scale environments, an improved path planning algorithm based on the integration of A* and DWA is proposed and applied to driverless ferry vehicles. Aiming at the traditional A* algorithm, the vector angle cosine value is introduced to improve the heuristic function to enhance the search direction; the search neighborhood is expanded and optimized to improve the search efficiency; aiming at the problem that there are many turning points in the A* algorithm, a cubic quasi-uniform B-spline curve is used to smooth the path. At the same time, fuzzy control theory is introduced to improve the traditional DWA so that the weight coefficient of the evaluation function can be dynamically adjusted in different environments, effectively avoiding the problem of a local optimal solution. Through the fusion of the improved DWA and the improved A* algorithm, the key nodes in global planning are used as sub-target punctuation to guide the DWA for local planning, so as to ensure that the ferry vehicle avoids obstacles in real time. Simulation results show that the fusion algorithm can avoid unknown dynamic and static obstacles efficiently and in real time on the basis of obtaining the global optimal path. In different environment maps, the effectiveness and adaptability of the fusion algorithm are verified.

## 1. Introduction

In recent years, with the rapid development of science and technology and the breakthrough of artificial intelligence technology, driverless cars have become a hot topic in the field of the automobile industry [1]. Driverless ferry vehicles have also been greatly developed on campuses. On many closed campuses, especially in large campus areas, many schools and enterprises are introducing or developing driverless ferry vehicles one after another [2]. Although a campus is not as complex as urban traffic conditions, it is still full of complex elements and uncertainties. At the same time, the campus scene puts forward higher requirements for the speed and safety of ferry vehicles [3]. Therefore, path planning is particularly important in the research and application of driverless ferry vehicles.

At present, the more recognized path planning methods in the industry can be divided into global path planning and local path planning [4]. Global path planning is used to plan a collision-free path according to the known environment map. Common planning algorithms include the Dijkstra algorithm [5], the A* algorithm [6], the RRT* algorithm [7], the ant colony algorithm [8], and so on. Local path planning is used to plan a collision-free optimal path from the local starting point to the local target point through the continuous acquisition of surrounding environment information by its own sensors. Common planning algorithms include the artificial potential field method [9], the TEB algorithm [10], the DWA [11], and so on. Both global and local path-planning algorithms have limitations. At present, many scholars have carried out a lot of research work in the field of path planning and improved and integrated the traditional path planning algorithm to improve its performance. For example, Tang et al. proposed to filter the redundant nodes in the A* algorithm and use cubic B-spline curves to optimize broken lines, which reduces the number of nodes and makes the overall path smoother, but it is not suitable for path planning in environments with dynamic obstacles [12]. Wu et al. integrate the APF algorithm with the RRT* algorithm and introduce the virtual field of APF into the search tree expansion phase of the RRT* algorithm, which improves the convergence rate and search efficiency of the RRT* algorithm, but it is only suitable for a simple experimental environment [13]. Dai et al. introduced the greedy algorithm into the RRT* algorithm, changed the search range of potential optimal parent nodes into a construction path rather than a node tree, which reduced the number of nodes to be searched, and then integrated the DWA to improve the efficiency of autonomous navigation, but the search mode with fixed parameters will inevitably fall into the local optimal state [14]. Shang et al. introduced an A* algorithm based on variable step size, which shortens the computing time of the algorithm, but it is only suitable for specific scenarios [15].

Based on the problems existing in the above research situation, this paper carries out the following work for the path planning algorithm of self-driving ferry vehicles: For the global planning method, in view of the low efficiency of the traditional A* algorithm, there are many turning points in the planned path, the vector angle cosine value is introduced to improve the heuristic function of the A* algorithm, and then the search neighborhood of the algorithm is extended and optimized. Finally, the cubic quasi-uniform B-spline curve is used to smooth the global path. For the local planning method, in order to solve the problem that the weight coefficient of the trajectory evaluation function of the traditional dynamic window method is fixed and the environmental adaptability is poor, fuzzy control theory is introduced to dynamically adjust the weight coefficient of the evaluation function of the traditional DWA. Finally, the two algorithms are fused, which solves the problems of the A* algorithm’s inability to avoid unknown obstacles and the DWA easily falling into local optimization in a large area environment, and the performance of the fusion algorithm is evaluated through simulation experiments.

## 2. Rasterization of the Environmental Map

Considering that the longitudinal height of the road can be ignored in the conventional ferry vehicle path planning experiment, the type of map used in the ferry vehicle’s path planning is an occupied grid map [16], which has accurate environmental modeling and can be updated in real time. The specific construction method is to rasterize the environment map and replace the environment map with several equal-sized square simulations to obtain a raster map. Converting continuous map data into discrete raster data has the advantages of simplifying data processing, providing convenient coordinate positioning, and adapting to different resolutions.

(1) Raster map binarization

In this article, the black grid is the known obstacle area, represented by the number “1”; the white grid is the drivable area, represented by the number “0”.

(2) How to mark grid positions

**Figure 1:**
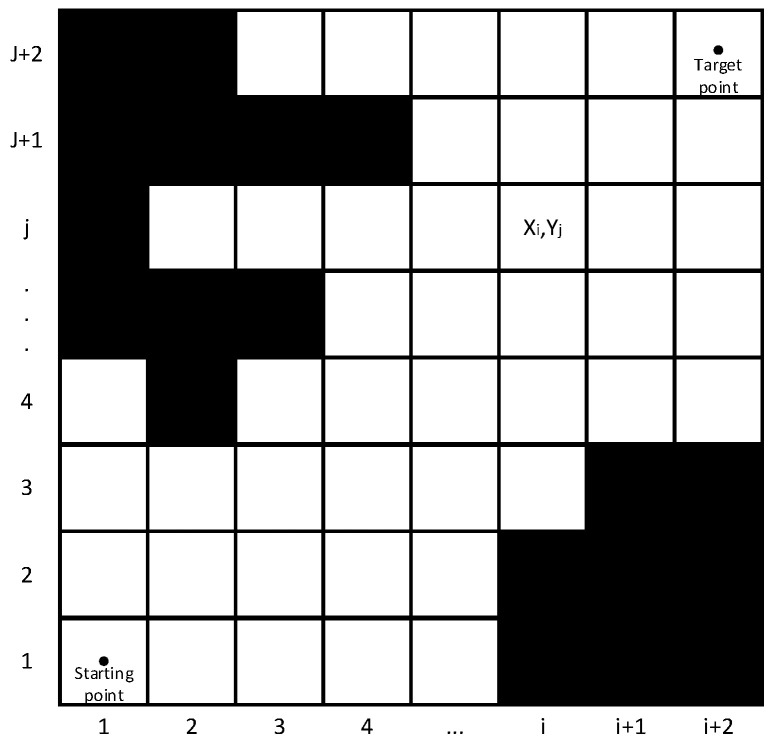
Raster map and raster location marker map.

A Cartesian rectangular coordinate system is established based on the two adjacent sides of the raster map, as shown in Figure 1. The grid position can be expressed as follows:

(1) $$\left(x_{i},y_{j})=[(i-1)*l+0.5l,(j-1)*l+0.5l\right]$$

In the formula: $x_{i}$ is the horizontal coordinate point; $y_{j}$ is the vertical coordinate point; l is the single-grid side length.

(3) Grid expansion processing

In order to ensure the safety of the ferry vehicles when driving, when generating a grid map, the obstacles whose areas do not meet a grid are expanded to fill a grid.

## 3. Global Path Planning

### 3.1. Traditional A* Algorithm

The A* algorithm is a typical heuristic search algorithm that is based on the Dijkstra algorithm and adds a heuristic function [17]. In addition to considering the actual cost, it also introduces the estimated cost from the target point to the current point, which is mainly used in static path planning when global environmental information is known. It guides the search and expansion of nodes through the evaluation function. The general expression of the evaluation function is as follows:

(2) F(n)=G(n)+H(n)

In the formula:

F(n) represents the total cost estimate from the starting point to the target point via node n. G(n) represents the actual substitution value from the starting point to node n. H(n) denotes the estimated generational value of node n to the target point. The distance cost is generally calculated by Euclidean distance, and the formula is as follows:

(3) $$G_{n}=\sqrt{{\left(x_{n}-x_{start}\right)}^{2}+{\left(y_{n}-y_{start}\right)}^{2}}$$

(4) $$H_{n}=\sqrt{{\left(x_{goal}-x_{n}\right)}^{2}+{\left(y_{goal}-y_{n}\right)}^{2}}$$

where $\left(x_{n},y_{n}\right)$ represents the current node coordinates, $\left(x_{start},y_{start}\right)$ represents the starting point coordinates, and $\left(x_{goal},y_{goal}\right)$ represents the target point coordinates.

The A* algorithm mainly selects the best path by maintaining the nodes in the Open and Close lists [18]. The specific implementation steps of the traditional A* algorithm are as follows:

(1) First, add the starting node S to the Open list, expand the sub-nodes of the 3 × 3 domain around the starting point, put the nodes that do not exist in the Close list and the non-obstacle nodes into the Open list, and delete the starting point from the Open list and put it into the Close list.
(2) Traverse the nodes in the Open list at this time, calculate the value of F(n) of each node according to the cost estimation function, and mark the node with the lowest value of F(n) as $S_{1}$.
(3) Take the $S_{1}$ node out of the Open list and add it to the Close list. Determine whether node $S_{1}$ is the target point; if it is the target point, then end the search and jump to step (6); the algorithm runs successfully; if not, perform the following steps:
(4) Search for a neighborhood node n that is reachable by node $S_{1}$ and does not exist in the Close list. If n already exists in the Open list, the values of G(n) for path 1$\left(S\to S_{1}\to n\right)$ and path 2 (S→n) are calculated. If the G(n) value of path 1 is less than the G(n) value of path 2, it is used as the parent node of n and its G(n) and its H(n) values are updated. If the value of path 1 is greater than the value of path 2, continue querying other nodes in the Open list. If n does not exist in the Open list, it is put into the Open list, and the cost is calculated. Record the node with the lowest F(n) value as new $S_{1}$.
(5) Return to step (3) until the target node is placed in the Close list or the Open list is empty.
(6) Starting from the target point, we continue to backtrack to find the final path of the plan.

### 3.2. Improved A* Algorithm

#### 3.2.1. Improved Heuristic Function

**Figure 2:**
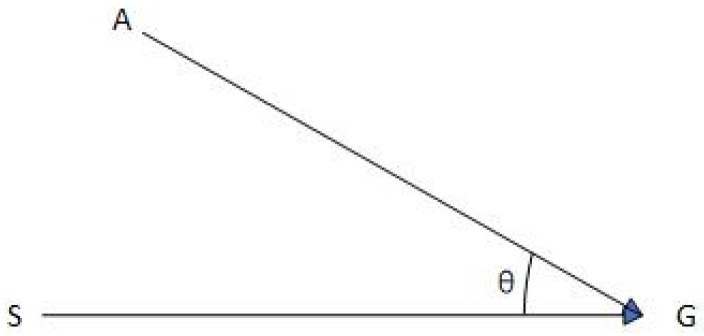
Vector diagram.

In order to reduce the inspection of useless nodes and make the path planning directional, the vector angle cosine value is introduced to improve the heuristic function, and the angle range is limited to 0 to 90 degrees. According to the nature of the cosine function, it monotonously decreases between 0 and 90 degrees. This ensures the monotonous constraint of the heuristic function and makes the selection of extended nodes more directional. The improvement steps are as follows:

(1) Build a vector $\vec{SG}$ from the start point to the target point and a vector $\vec{AG}$ from the extension node of the current node to the end node, as shown in Figure 2. Calculate the angle cosine cosθ between the two vectors, as shown in Formula (5):

(5) $$\cos\theta =\frac{\vec{AG}•\vec{SG}}{\left|\vec{AG}\right|•\left|\vec{SG}\right|}$$

(2) Filter out the extended nodes whose cosine value is less than 0.5, make the angle of vector $\vec{SG}$ and vector $\vec{AG}$ less than 60 degrees, and ensure that the search path is closer to the destination.
(3) Use η as the weight and adjust according to the actual size of the map, which is the map unit length multiplied by the resolution. The resolution refers to how many grids the 1 m unit length consists of. The following raster map 1 m is a grid, so η=1. The constructor is used as an evaluation function, as shown in Formula (6):

(6) $$H_{1}(n)=H(n)-\eta \cos\theta$$

Because the cosine function is a nonlinear monotone function, it can effectively restrict the search node range of the A* algorithm to a certain range. It can better reduce the number of useless nodes and improve the search efficiency of the A* algorithm.

The new heuristic functions obtained from Formulas (5) and (6) are as follows:

(7) F(n)=G(n)+H(n)−ηcosθ

**Figure 3:**
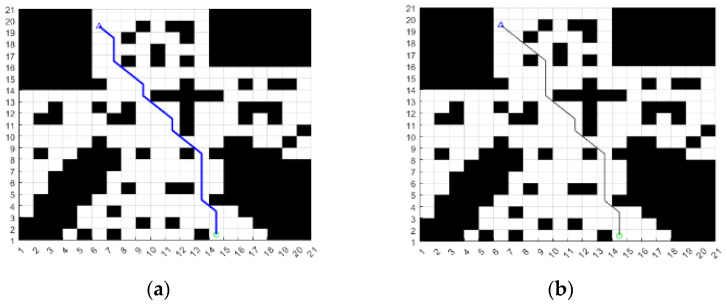
Comparison of heuristic functions before and after the improved A* algorithm. (**a**) Traditional A*; (**b**) improved A* of the heuristic function.

The effectiveness of the improved A* algorithm before and after the heuristic function is verified by simulation; the starting point and the end point are set in the same grid map, and the programs are written to run the improved A* algorithm, respectively. The path-planning results are shown in Figure 3.

Statistics of the planning results and a summary of the relevant data of the planning path are shown in Table 1.

From the path in Figure 3 and the data in Table 1, we can see that the A* algorithm with an improved heuristic function has fewer path inflection points and a more directional search, and the path length and planning time have more advantages than the traditional A* algorithm.

#### 3.2.2. Expand the Search Neighborhood

The traditional A* algorithm needs to traverse the map nodes when planning the path. The more nodes are traversed, the longer the search time is. When the environmental map is large, the traditional A* algorithm will traverse many nodes on the map when planning the path, resulting in a long search time, and the planned path is not smooth enough to meet the planning needs of the ferry vehicles. The number of nodes included in the Close list of the algorithm is proportional to the planning time of the algorithm. The more nodes are included, the longer the planning time is, so the planning time of the algorithm can be reduced by reducing the number of nodes in the Close list. Therefore, this section reduces the number of nodes in the Close list by expanding the search neighborhood.

**Figure 4:**
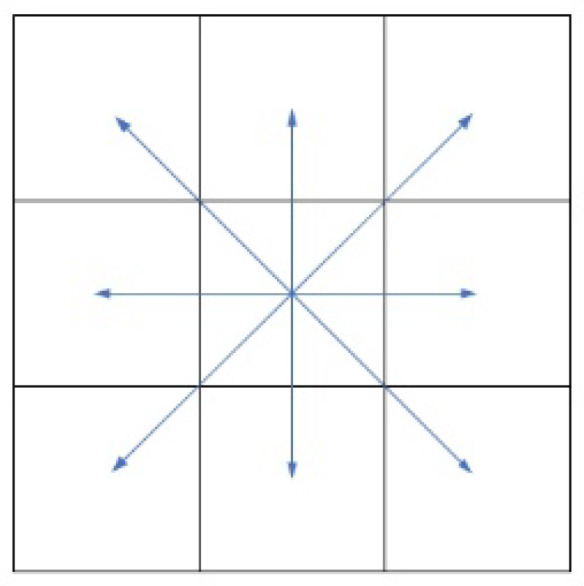
Search the neighborhood with 3 × 3.

The traditional A* algorithm searches the grid of the 3 × 3 neighborhood around the current node each time, which is shown in Figure 4.

**Figure 5:**
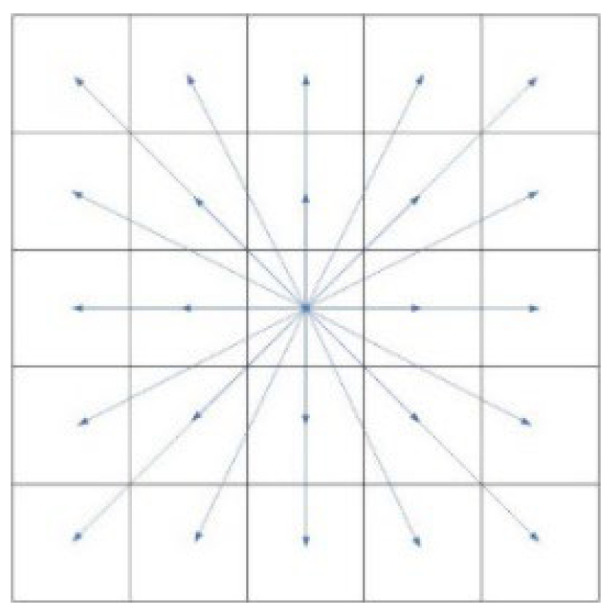
Search the neighborhood with 5 × 5.

The traditional 3 × 3 search neighborhood will explore the eight adjacent nodes except the current point each time, select one of the eight adjacent nodes as the optimal node according to the estimated function value, and then repeat the above steps. In this way, the algorithm can only explore eight adjacent nodes at a time, the direction of the planning path is also limited to an integer multiple of π/4, the planning time is longer, and there are more turning points in the planning path. In order to solve this problem, this paper extends the search neighborhood of the traditional algorithm from 3 × 3 to 5 × 5 to reduce the number of nodes included in the Close list, increase the searchable direction of the planned path, and improve the smoothness of the path. The 5 × 5 search neighborhood diagram is shown in Figure 5.

**Figure 6:**
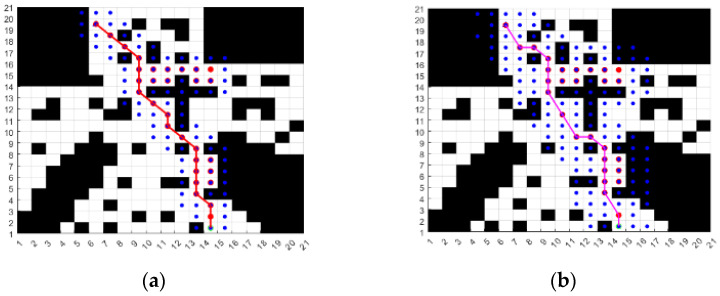
Schematic diagram of the planning result. The lines represent the searched paths and the circles represent the searched nodes. (**a**) Search the neighborhood with 3 × 3; (**b**) search the neighborhood with 5 × 5.

As can be seen from Figure 5, the scope of the 5 × 5 search neighborhood is wider, and the number of adjacent nodes in each search increases from 8 to 24, which is three times higher than that of the traditional A* algorithm, and 1 of every 24 neighboring nodes is included in the Close list. Compared with the traditional algorithm, the number of nodes included in the Close list can be effectively reduced, and the search time of the algorithm will be reduced. At the same time, the searchable direction of each traversal process of the algorithm is also improved; the search direction rises from 8 to 16, the turning angle of the path in the search step becomes smaller, the sharp nodes in the planning path can be reduced, and the smoothness of the path can be improved accordingly. The effectiveness of the 5 × 5 search neighborhood A* algorithm is verified by simulation, and the starting point and end point are set. Based on the improved heuristic function, the A* algorithm is programmed to search the neighborhood with 3 × 3 and 5 × 5, respectively, and the path planning results are shown in Figure 6. The blue dots in the figure are nodes that have been traversed.

Statistics the planning results and summarize the relevant data of the planning path into Table 2.

As can be seen from the paths in Figure 6 and the data in Table 2, the number of nodes collected in the neighborhood of 3 × 3 search is 31, the length of the planning path is 21.77 m, and the planning time is 0.87 s, while the number of nodes in the 5 × 5 search neighborhood is 29, the length of the planning path is 21.31 m, and the planning time is 0.74 s. It can be seen that the length and time of the planning path in the 5 × 5 search neighborhood are better than those in the 3 × 3 search neighborhood, and the planned path is smoother. Therefore, it can be concluded that the method of expanding the search neighborhood can effectively improve the planning efficiency and path quality of the algorithm.

**Figure 7:**
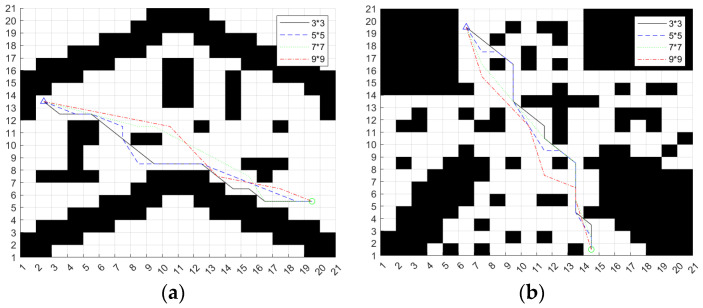
Results of different searches for neighborhood planning. (**a**) Map 1; (**b**) Map 2.

Because of the large area of the campus scene map and more grids, we can expand the larger search neighborhood to plan the path. In the same way, the A* algorithm based on the improved heuristic function extends the 7 × 7 search neighborhood and the 9 × 9 search neighborhood, respectively, and selects two different maps to verify the algorithm. The search results of different search neighborhoods are shown in Figure 7.

Summarize the different search neighborhood planning results of Map 1 in Table 3.

Summarize the different search neighborhood planning results of Map 2 in Table 4.

From the planning results in Table 3 and Table 4, we can see that the path planning results of the algorithm in different search neighborhoods are also quite different. In the two maps, the path length and planning time of the 9 × 9 search neighborhood are the best; there are more grids in the 9 × 9 search neighborhood, so it will reduce the search time of the algorithm, and the overall planning path is smoother. Therefore, this paper uses a 9 × 9 search neighborhood as the scheme to improve the A* algorithm.

#### 3.2.3. Smoothing Treatment

After improving the heuristic function and expanding the search neighborhood optimization, the A* algorithm has seen a significant improvement in the planning efficiency and quality of the planned path, but the planned path still has the problem of a large number of right-angle turning times. This turning point does not meet the vehicle kinematics constraints and is not suitable for vehicle trajectory tracking control. Therefore, the cubic quasi-uniform *B*-spline curve is used to smooth the path planned by the improved A* algorithm [19].

The mathematical expression of the *B*-spline curve is as follows:

(8) $$P(u)=\sum_{i=0}^{n}P_{i}B_{i,k}(u)$$

In the formula, $P_{i}(i=0,1,2,\ldots ,n)$ is the control point and $\left\{B_{i,k}(u)\}(i=0,1,2,\ldots ,n\right\}$ is the *k*-th *B*-spline basis function.

The basis function is usually defined by de Boor−Cox recursion [20]. The principle is to construct a recursive formula, which is constructed by low and high order. The expression is as follows:

(9) $$B_{i,k}(u)=\left\{\begin{array}{l} \left\{\begin{array}{l} 1,u_{i}\leq u\leq u_{i+1}\\ 0,other \end{array}\right.\;k=1\\ \frac{u-u_{i}}{u_{i+k-1}-u_{i}}B_{i,k-1}(u)+\frac{u_{i+k}-u}{u_{i+k}-u_{i+1}}B_{i+1,k-1}(u),\;k\geq 2\\ \frac{0}{0}=0 \end{array}\right.$$

In the formula, $\left\{B_{i,k}(u)\right\}$ denotes the *i*-th *k*-th *B*-spline basis function, $u_{i}$ represents the *i*-th parameter point, and specifies 0/0=0. It is known from the above formula that the *B*-spline basis function can be solved by spline degree *k* and node vector U. In this paper, the cubic quasi-uniform *B*-spline curve is selected to smooth the planned path, and the control point can be set to triple. The basis function of a cubic quasi-uniform *B*-spline curve is obtained, as shown in Formula (10).

(10) $$\left\{\begin{array}{l} b_{0}=\frac{1}{6}(-u^{3}+3u^{2}-3u+1)\\ b_{1}=\frac{1}{6}(3u^{3}-6u^{2}+4)\\ b_{2}=\frac{1}{6}(-3u^{3}+6u^{2}+3u+1)\\ b_{3}=\frac{1}{6}u^{3} \end{array}\right.$$

Therefore, the equation of a cubic quasi-uniform *B*-spline curve is Equation (11):

(11) $$P_{0,3}(u)=\frac{1}{6}\left[1\;u\;u^{2}\;u^{3}\right]\left[\begin{array}{cccc} 1 & 4 & 1 & 0\\ -3 & 0 & 3 & 0\\ 3 & -6 & 3 & 0\\ -1 & 3 & -3 & 1 \end{array}\right]\left[\begin{array}{l} P_{0}\\ P_{1}\\ P_{2}\\ P_{3} \end{array}\right]$$

In the formula, u is the position parameter and $P_{0}-P_{3}$ is the control point.

**Figure 8:**
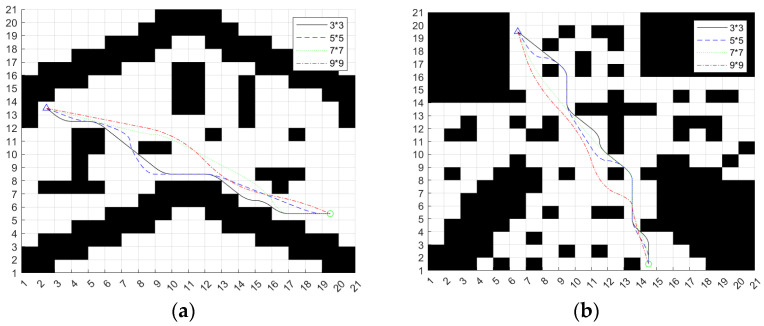
Smoothed path. (**a**) Map 1; (**b**) Map 2.

The cubic quasi-uniform *B*-spline curve is used to deal with the path planned in Section 3.2.2, Figure 7, and the result is shown in Figure 8.

As can be seen from Figure 8, the curvature of the smooth path is continuous, which can meet the trajectory tracking requirements of the ferry vehicle.

The final improved A* algorithm is used to first introduce the vector angle cosine value into the heuristic function of the algorithm to improve the direction of the algorithm, then expand the search neighborhood to improve the search efficiency of the algorithm, and finally smooth the path through the cubic quasi-uniform *B*-spline curve, improving path quality.

## 4. Local Path Planning

### 4.1. Traditional DWA

**Figure 9:**
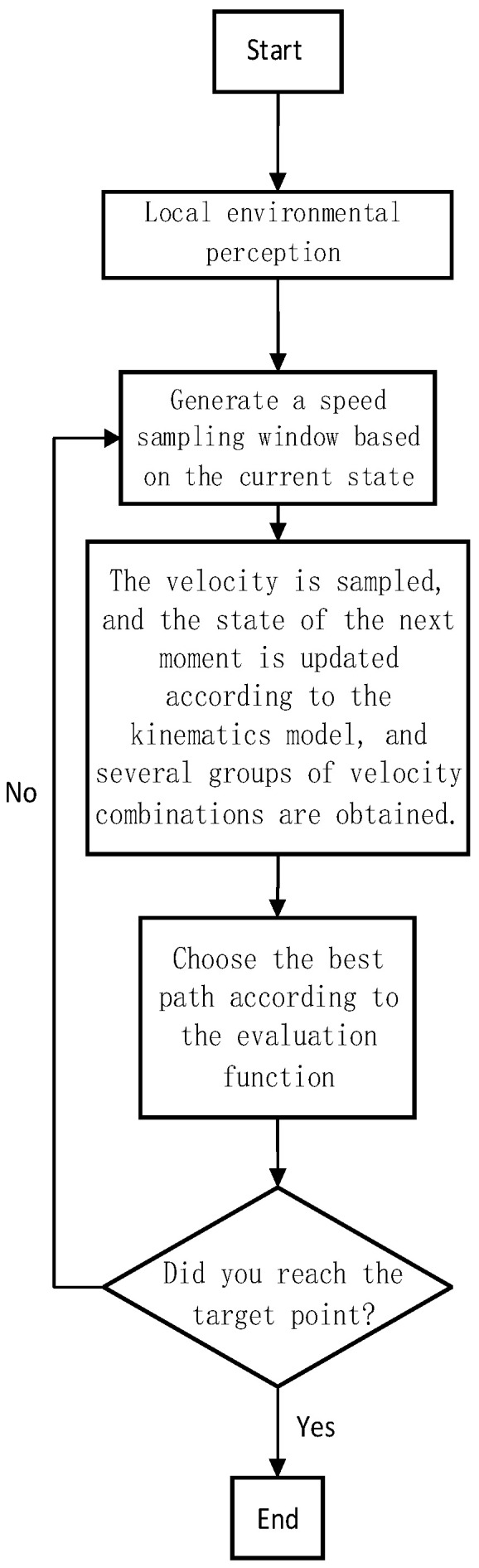
Flow chart of DWA algorithm.

DWA stands for dynamic window algorithm. On the basis of considering the environmental information, kinematic model, and kinematic performance of the ferry vehicle, the DWA samples several groups of velocity combinations (v,ω) in a simulation period from the allowable linear velocity $\left[v_{\min},v_{\max}\right]$ and angular velocity $\left[\omega_{\min},\omega_{\max}\right]$, and simulates the motion trajectories of each group of sampling speed combination (v,ω). Then, according to the trajectory evaluation function, the trajectory evaluation value of each group of velocity combinations is calculated, from which the linear velocity $v_{best}$ and angular velocity $\omega_{best}$ corresponding to the optimal trajectory are selected, and the obtained velocity combination $\left(v_{best},\omega_{best}\right)$ is used as the speed instruction in the current motion cycle. Cycle the above steps until you reach the target point. The dynamic window method can be divided into three parts: motion model, velocity sampling space, and trajectory evaluation function [21]. The flow chart of the DWA is shown in Figure 9.

#### 4.1.1. Motion Model of a Driverless Ferry Vehicle

**Figure 10:**
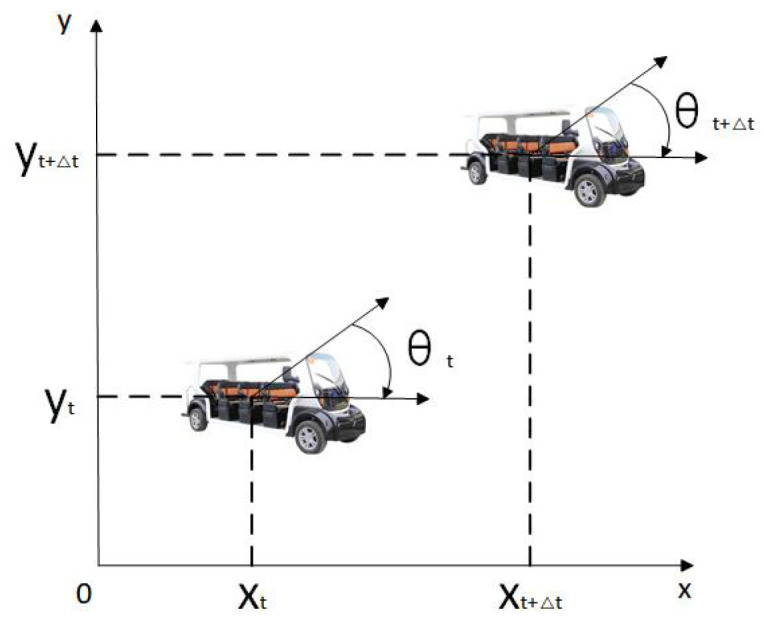
The sports degree posture of the driverless ferry vehicle.

First of all, we need to establish a mathematical model to simulate the trajectory information of the driverless ferry vehicle. The kinematic model of the driverless ferry vehicle [22] is shown in Figure 10.

Suppose that in Δt time, the ferry bus can be regarded as a uniform linear motion to simulate the ferry movement, and the trajectory is calculated according to one group of state values $\left(v_{t},\omega_{t}\right)$, and its motion model is as follows:

(12) $$\left[\begin{array}{l} x_{t+\Delta t}\\ y_{t+\Delta t}\\ \theta_{t+\Delta t} \end{array}\right]=\left[\begin{array}{l} x_{t}\\ y_{t}\\ \theta_{t} \end{array}\right]+\left[\begin{array}{ccc} \cos\theta & -\sin\theta & 0\\ \sin\theta & \cos\theta & 0\\ 0 & 0 & 1 \end{array}\right]\left[\begin{array}{l} v_{x}\Delta t\\ v_{y}\Delta t\\ \omega_{t}\Delta t \end{array}\right]$$

In the formula: ${\left[x_{t+\Delta t}\;y_{t+\Delta t}\;\theta_{t+\Delta t}\right]}^{T}$ is the position of the ferry vehicle in the t+Δt-time world coordinate system; ${\left[x_{t}\;y_{t}\;\theta_{t}\right]}^{T}$ is the posture of the ferry vehicle in the t time world coordinate system; ${\left[v_{x}\Delta t\;v_{y}\Delta t\;\omega_{t}\Delta t\right]}^{T}$ is the position and attitude change under the t time ferry bus coordinates.

#### 4.1.2. Driverless Ferry Vehicle Speed Sampling Space

**Figure 11:**
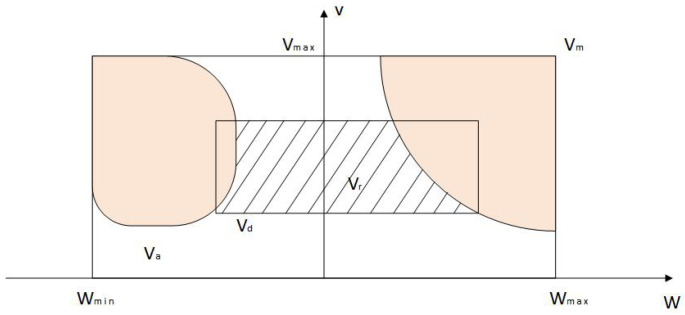
Speed sampling space of ferry vehicles.

In the actual driving process of the vehicle, its speed is related to the inherent mechanisms of the vehicle, such as the motor and brake. At the same time, the obstacles and road boundaries in the driving environment also limit the speed, so in the actual speed sampling process, it is necessary to sample in the speed window that meets certain constraints. The speed sampling space diagram of the vehicle during driving is shown in Figure 11.

Combined with the above figure, the speed sampling space is analyzed, and the conditions that limit the vehicle speed and angular speed mainly include the following:

(1) Kinematic constraint

Affected by dynamic performance and other key factors, the expression of the maximum and minimum speed range of the vehicle is as follows:

(13) $$V_{m}=\left\{(v,\omega )|v_{\min}\leq v\leq v_{\max},\omega_{\min}\leq \omega \leq \omega_{\max}\right\}$$

Among them, $v_{\min}$ and $v_{\max}$ represent the minimum speed and maximum speed of the vehicle, respectively, in units of m/s. $\omega_{\min}$ and $\omega_{\max}$ represent the minimum angular speed and maximum angular speed of the vehicle, respectively, in units of rad/s.

(2) Dynamic constraint

The vehicle is limited by the performance of the acceleration and braking mechanisms [23]. When the vehicle accelerates or brakes at maximum linear acceleration or turns at maximum angular acceleration or deceleration, the dynamic speed sampling space of the vehicle can be obtained as shown in the following formula:

(14) $$V_{d}=\left\{(v,\omega )|v_{c}-{\dot{v}}_{b}\Delta t\leq v\leq v_{c}+{\dot{v}}_{a}\Delta t,\omega_{c}-{\dot{\omega }}_{b}\Delta t\leq \omega \leq \omega_{c}+{\dot{\omega }}_{a}\Delta t\right\}$$

where $v_{c}$ is the frontline speed of the vehicle, in units of m/s; ${\dot{v}}_{a}$ and ${\dot{v}}_{b}$ are the maximum acceleration and deceleration of the vehicle in the course of driving, in units of ${m/s}^{2}$. $\omega_{c}$ is the current angular speed of the vehicle, in rad/s units; ${\dot{\omega }}_{a}$ and ${\dot{\omega }}_{b}$ are the maximum angular acceleration and deceleration of the vehicle, in units of ${\mathrm{rad}/s}^{2}$.

(3) Security constraint

In order to ensure the safety of the vehicle, it is necessary to control the vehicle to stop moving before the nearest obstacle collides, so the speed range is further reduced to Formula (15):

(15) $$V_{a}=\left\{(v,\omega )|v\leq \sqrt{2\cdot dist(v,\omega )\cdot {\dot{v}}_{b}},\omega \leq \sqrt{2\cdot dist(v,\omega )\cdot {\dot{\omega }}_{b}}\right\}$$

In the formula, dist(v,ω) is the nearest distance to the obstacle on the corresponding trajectory of the velocity vector (v,ω).

Combining the above three speed constraints, the final selectable velocity sampling space is the intersection of the above three sets, so that $v_{r}$ represents the allowable velocity set; then, $v_{r}$ should satisfy the following:

(16) $$v_{r}=v_{m}\cap v_{d}\cap v_{a}$$

**Figure 12:**
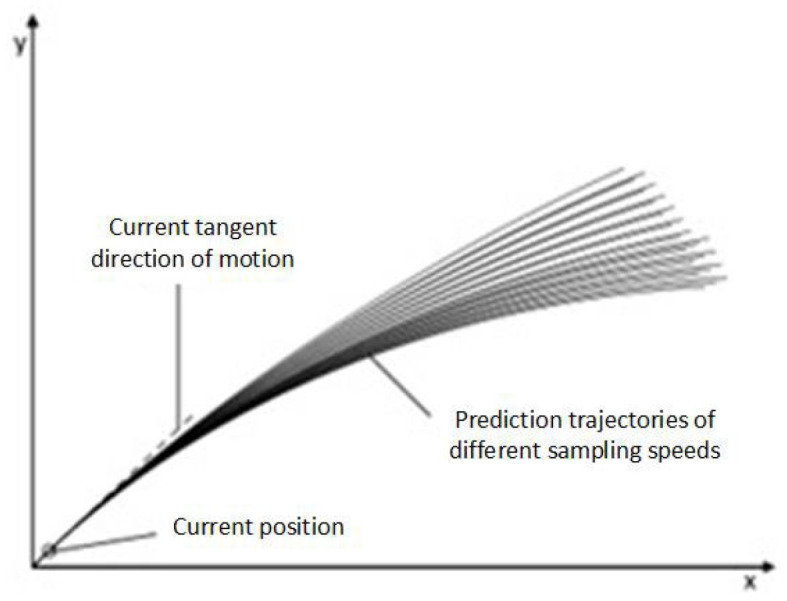
Trajectory prediction map.

As shown in Figure 11 above, $v_{r}$ is the intersection of the above different speed ranges. When the vehicle carries out speed sampling, it can only sample in the shadow space, that is, $v_{r}$ areas. In the continuous speed space $v_{r}$, (v,ω) is converted into discrete points according to the sampling points of the speed. The trajectory of the sampling points is predicted according to the dynamic characteristics of the vehicle, and multiple predicted trajectories at the next time, as shown in Figure 12.

The vehicle will score the multiple paths generated by the above image based on the trajectory evaluation function and select the optimal track for output.

#### 4.1.3. Trajectory Evaluation Function

Many tracks generated in the current speed sampling space are drivable paths for vehicles, but vehicles are usually expected to follow the most ideal path in actual driving. The optimal driving path should meet the following requirements:

(1) The planned path can reach the target point.
(2) The planned path should be kept at a safe distance from obstacles.
(3) The speed of the vehicle should be kept at a high level within the allowable range.

Based on the above requirements, the evaluation function of the traditional DWA is shown in Formula (17):

(17) G(v,ω)=α⋅heading(v,ω)+β⋅dist(v,ω)+γ⋅velocity(v,ω)

In the formula, heading(v,ω) is the heading angle evaluation factor between the different track points and the target points of the vehicle in the current planning cycle, and the calculation formula is shown in Formula (18):

(18) $$heading(v,\omega )=180^{\circ }-\theta$$

In the formula, θ is the angle between the end of the predicted track of the vehicle and the connection line of the target point, in degrees. The smaller the value of θ is, the more inclined the mobile robot is to move towards the target point, and the larger the target azimuth evaluation function is.

dist(v,ω) is the evaluation factor of the distance between the different tracks of the vehicle and the nearest obstacle in the current planning cycle, as shown in Formula (19):

(19) $$dist(v,\omega )=\left\{\begin{array}{l} d,\;d<d_{\max}\\ d_{\max},\;d\geq d_{\max} \end{array}\right.$$

In the formula, d is the distance from the point on the track of the vehicle to the nearest obstacle, and $d_{\max}$ is the threshold of the distance from the vehicle to the obstacle. The larger the distance evaluation factor is, the farther the vehicle is from the obstacle on the predicted trajectory, and the higher the safety of the vehicle. The function of the formula $d_{\max}$ is to ensure that there are no obstacles on a certain simulation trajectory, which leads to a too large obstacle distance evaluation factor and a too large proportion in the total trajectory evaluation function.

velocity(v,ω) is the speed evaluation factor of the vehicle in the current planning cycle, as shown in Formula (20):

(20) $$velocity(v,\omega )=abs(v_{s})$$

In the formula, abs represents the absolute value of the parameter, and $v_{s}$ is the linear speed corresponding to the predicted track of the vehicle, in units of m/s. The higher the value of the speed evaluation factor, the faster the vehicle can approach the target point on the simulation track, but too much speed will also cause the vehicle to miss the target point when it is very close to the target point.

α, β, and γ represent the weight coefficients of different evaluation factors, respectively, and the range of values is [0,1]. Because the dimensions of the three evaluation factors are different, in order to avoid the excessive impact of one evaluation factor on the results, thus weakening the role of other evaluation factors, it is necessary to normalize the above evaluation factors. The normalized calculation formulas for different evaluation factors are shown in the following Formulas (21) to (23):

(21) $$normal(heading(i))=\frac{heading(i)}{\sum_{i=1}^{n}heading(i)}(i=1,2,\ldots ,n)$$

(22) $$normal(dist(i))=\frac{dist(i)}{\sum_{i=1}^{n}dist(i)}(i=1,2,\ldots ,n)$$

(23) $$normal(velocity(i))=\frac{velocity(i)}{\sum_{i=1}^{n}velocity(i)}(i=1,2,\ldots ,n)$$

In the formulae, n is the total number of predicted trajectories in the current velocity sampling space, i is the current predicted trajectory to be evaluated, and normal represents the normalized parameters. In summary, by calculating the value of the trajectory evaluation function of each predicted trajectory, the track with the highest score is selected, that is, the optimal trajectory.

#### 4.1.4. Problems with the DWA

Through the above analysis of the principle of the DWA, we can see that the selection of the optimal trajectory is realized by adjusting the three weight coefficients of the trajectory evaluation function. The traditional DWA uses a fixed weight coefficient for path planning, but it is obviously not appropriate to use a constant weight coefficient of the evaluation function for path planning in different environments, so how to determine the values of α, β, and γ is very important. At present, there is no detailed reference on how to select the weight coefficients α, β, and γ. In order to make the driverless ferry vehicle complete the autonomous navigation task in the complex dynamic scene, the local path planning algorithm needs to have certain flexibility. For the driverless ferry vehicle that uses the traditional dynamic window method for local path planning, if the weight coefficient in the trajectory evaluation function is constant, the navigation ability in the complex dynamic scene will decline, and the quality of the planned path will not be high [24]. In this paper, the problems and defects of the traditional DWA are analyzed through simulation experiments.

**Figure 13:**
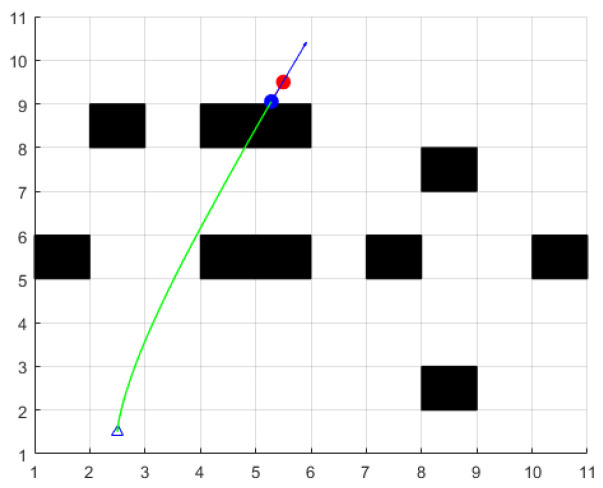
Scene one.

The setting parameters for the environment and ferry vehicle are as follows: the map size is 10 × 10, the obstacles are represented by black squares, the triangles represent the starting points, and the red circle represent the target points, and the blue circle represents the current position. The planned path is represented by a green line. The maximum speed of the ferry is 2 m/s, the maximum angular velocity is 30 deg/s, the maximum acceleration is $0.3\;{m/s}^{2}$, the maximum angular acceleration is $50\;\deg/s^{2}$, the velocity resolution is 0.01 m/s, the angular velocity resolution is 1 deg/s, the unit forward simulation time is $d_{t}=3\;s$, and the time resolution is 0.1 s. The above parameters of the ferry vehicle are consistent in the following simulation environment: Through the simulation experiment, it was found that the traditional dynamic window method has the following problems:

When the heading angle weight coefficient α is larger, the obstacle distance weight coefficient β is smaller, and the speed weight coefficient γ is smaller, the ferry vehicle will be more inclined to move towards the target point, but because it does not pay enough attention to obstacle avoidance, when the obstacle is on the line between the ferry vehicle and the target point, the vehicle cannot get around the obstacle very well, as shown in Figure 13. At the same time, α=0.8, β=0.1, and γ=0.1.

**Figure 14:**
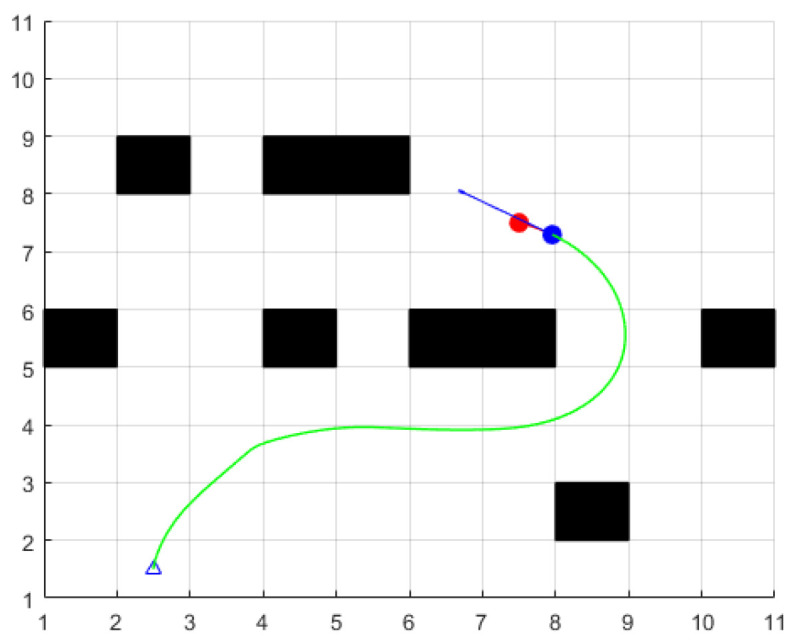
Scene two.

When the distance weight coefficient β of the obstacle is larger and the heading angle weight coefficient α and the speed weight coefficient γ are smaller, the ferry vehicle will be more inclined to bypass the obstacle. But this may cause the ferry vehicle to avoid the obstacles prematurely, thus preventing the vehicle from passing through the free space between the two obstacles, choosing to bypass the obstacles and increasing the length of the path, as shown in Figure 14; at the same time, α=0.1, β=0.8, and γ=0.1.

**Figure 15:**
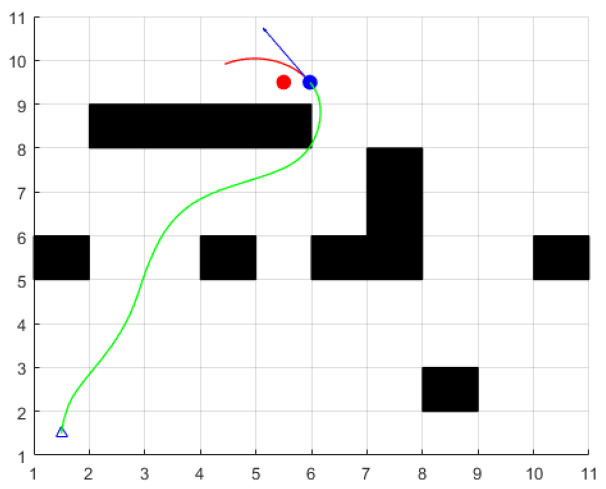
Scene three.

When the speed weight coefficient γ is larger and the heading angle weight coefficient α and the obstacle distance weight coefficient β are smaller, the ferry vehicle will be more inclined to choose the track with high speed, but if the speed is too fast, the ferry vehicle will not be able to slow down in time and will miss the target point when approaching it. As shown in Figure 15, at this time, α=0.1, β=0.1, and γ=0.8.

Thus, it can be seen that in different scenarios, the traditional dynamic window method cannot successfully complete the planning task because of its poor adaptability to the environment because of the fixed weight coefficient.

### 4.2. Improved DWA

Through the theoretical research and experimental verification of the traditional DWA, it can be found that the working environment of the DWA is diverse, and it is difficult to determine a set of appropriate fixed weight coefficients. The fixed weight combination in different scenes will seriously affect the path planning effect of the traditional DWA and may lead to problems such as the ferry vehicle not being able to avoid obstacles, the path being too long or missing the target point, and so on. In order to improve the above problems, this paper introduces fuzzy control theory to improve the traditional DWA to make it output the weight coefficient of the evaluation function adaptively according to the actual situation and improve the adaptability of the algorithm to the complex environment.

#### 4.2.1. Basic Theory of Fuzzy Control

Fuzzy control fuzzifies the input, and then inference and output through fuzzy rules, so as to realize the control of the system. The fuzzy control system has strong anti-interference ability and robustness, and the control effect is less affected by the change of parameters. At the same time, in the process of application, it is not required to establish an accurate mathematical model for the controlled object, and the control strategy is relatively simple and convenient for application [25].

**Figure 16:**
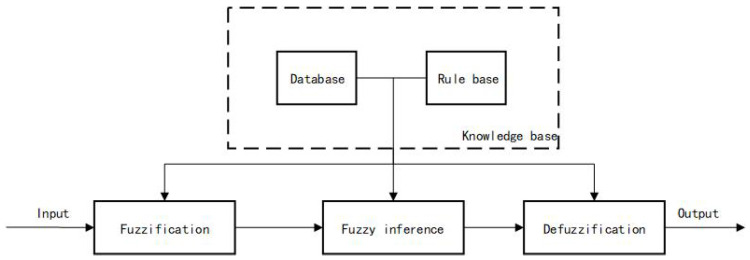
Fuzzy controller framework.

The design of a fuzzy controller mainly includes four parts: input fuzzification, establishment of fuzzy control rules, fuzzy inference, and defuzzification, of which the core part is the establishment of fuzzy control rules. The design principle of the fuzzy controller framework is shown in Figure 16.

#### 4.2.2. Design of a DWA Based on Fuzzy Control

According to the characteristics of the DWA and the basic principle of the fuzzy controller, we can know that the ideal output value can be obtained when selecting the appropriate input value, selecting the reasonable membership function, and formulating the appropriate fuzzy control rules. The effect of path planning in the traditional DWA mainly depends on three weight coefficients. Therefore, this paper uses fuzzy control theory, considers the directionality principle and safety principle in path planning, and introduces the weight coefficient to design three fuzzy controllers, respectively, to realize the dynamic adjustment of the three weight coefficients of α, β, and γ. In order to improve the adaptability of the ferry in a dynamic environment.

(1) Fuzzification

**Figure 17:**
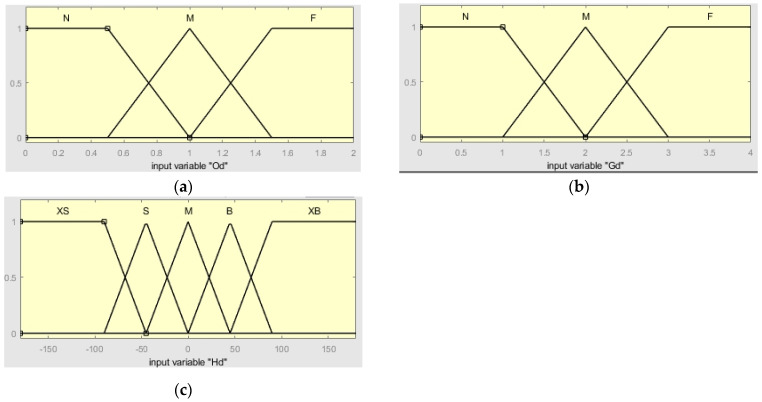
Od, Gd and Hd membership function. (**a**) Od membership function; (**b**) Gd membership function; (**c**) Hd membership function.

In the fuzzy controller designed in this section, the inputs involved are the distance between the ferry vehicle and the obstacle Od; the distance between the ferry vehicle and the target point Gd; the heading angle of the ferry vehicle relative to the target point Hd; the output includes the three weight coefficients of the DWA α, β, and γ; and the fuzzy rule weight coefficient ε. The fuzzy definition of input and output is as follows: The fuzzy set of Od is defined as {near (N), medium (M), far (F)}, and the domain is [0, 2]. When Od≤0.5, it is fuzzified to N; when Od=1, it is fuzzified to M; and when Od≥1.5, it is fuzzified to F. The fuzzy set of Gd is {near (N), middle (M), far (F)}, and the domain is [0, 4]. When Gd≤1, it is fuzzified to N; when Gd=2, to M; when Gd≥3, to F. The unit of Hd is degree (deg), the fuzzy set is {negative large (XS), negative (S), medium (M), positive (B), positive large (XB)}, and the domain is [−180, 180]. When Hd≤−90, the fuzzy description is negative large (XS); when Hd≥90, the fuzzy description is positive large (XB). When Hd=−45, fuzzy description is negative (S); when Hd=45, fuzzy description is positive (B); and when Hd=0, fuzzy description is medium (M). Figure 17 shows the membership functions of Od, Gd, and Hd.

**Figure 18:**
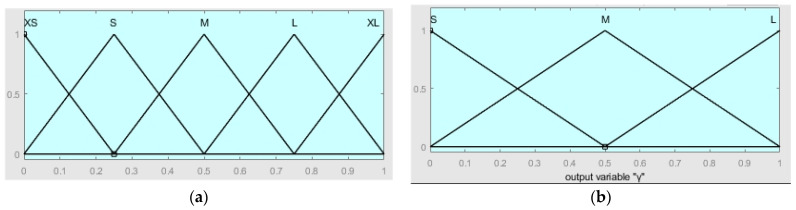
Membership function of output. (**a**) α, β, and ε membership function; (**b**) γ membership function.

For the output of the fuzzy controller α, β, and γ, the fuzzy set is {XS, small (S), medium (M), large (L), and maximum (XL)}, and the domain is [0, 1]. For the output of γ, the domain is [0, 1]. The fuzzy set is {small (S), medium (M), large (L)}, as shown in Figure 18, is the membership function of the output.

(2) The formulation of fuzzy rules

**Figure 19:**
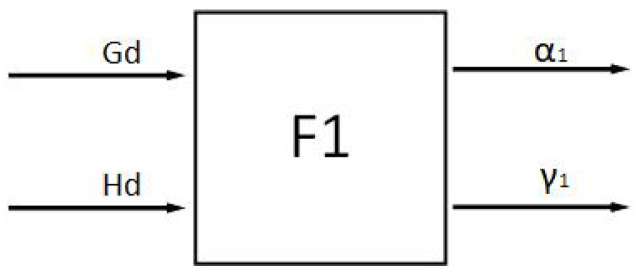
Directional fuzzy controller.

Fuzzy rules are the core of fuzzy controllers [26]. According to the analysis of the traditional DWA, the adjustment of the weight coefficient of the DWA is actually a trade-off between the direction, safety, and speed of the ferry vehicle according to the current environment, so as to find out the most suitable speed trajectory for the current target. Among the three principles, the driving speed will have an impact on the algorithm efficiency of both directivity and safety principles. Therefore, this paper first designs two fuzzy controllers considering the directionality principle and the safety principle, and then designs a fuzzy controller that can output the weight coefficients of the directivity and safety principles, thus obtaining the weight coefficients of different evaluation factors in the evaluation function of the DWA. The specific fuzzy rules are designed as follows:

The factors that affect the directionality principle are the distance Gd between the ferry vehicle and the target point and the navigation angle Hd with the target point, so the structure of the directional fuzzy controller $F_{1}$ is shown in Figure 19.

When the ferry vehicle approaches the target point and the heading angle is large, the direction should be given priority, a larger value of α should be used, and the speed should be reduced to a smaller value of γ. When the distance between the ferry vehicle and the target point is long, and the heading angle is small, priority should be given to increasing the driving speed, using a larger value of γ, and choosing a moderate value of α to keep moving in the direction of the target. When it is far away from the target point, priority is given to increasing the speed and then gradually maintaining the heading angle close to the target point. When approaching the target point, gradually reduce the speed and give priority to the direction. The specific fuzzy rules are shown in Table 5.

After the fuzzy vector under the directional principle is obtained by the Mamdani type reasoning method [27], the clear output control quantity is obtained by defuzzifying by the center of gravity method, and the accurate values of α and γ can be obtained. At the same time, in order to take into account for the security, the value of $\beta_{1}$ is 0.2.

**Figure 20:**
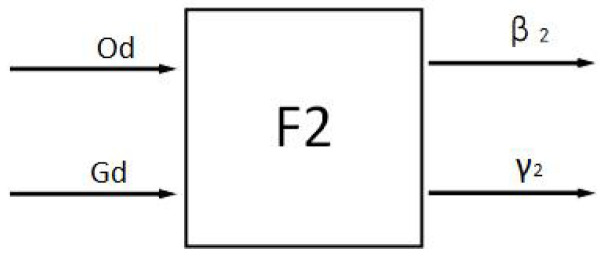
Safety fuzzy controller.

The factors affecting the safety principle are the distance Od between the ferry vehicle and the obstacle and the distance Gd from the target point, so the structure of the safety fuzzy controller $F_{2}$ is shown in Figure 20.

When the distance between the ferry vehicle and the obstacle and the target point is very small, the speed should be reduced first and the direction should be given priority. When the ferry vehicle is close to the target point and far from the obstacle, it should appropriately increase the speed, reduce the value of β, and give priority to approaching the target point. When the ferry vehicle is far from the obstacle and the target point, the ferry vehicle gives priority to speeding up close to the target point, and the requirement for obstacle avoidance is reduced. When the ferry vehicle is far away from the target point but close to the obstacle, we should give priority to avoiding obstacles, take a larger value for β, and reduce the speed by γ value appropriately. The specific security fuzzy rules are shown in Table 6. The output is defuzzified by the center of gravity method, and the exact values of output β and γ under the safety principle are obtained. At the same time, in order to take into account the directional principle, the value of $\alpha_{2}$ is 0.2.

Because different environments attach different importance to the directionality principle and safety principle, it is necessary to consider the distance Od between the ferry vehicle and the obstacle and the heading angle Hd with the target point and introduce the fuzzy rule weight coefficient ε of directionality and safety. By combining the output of the fuzzy controller obtained from $F_{1}$ and $F_{2}$, the final expressions of α, β, and γ are obtained:

(24) $$\left[\alpha ,\beta ,\gamma ]=(1-\varepsilon )[\alpha_{1},\beta_{1},\gamma_{1}]+\varepsilon [\alpha_{2},\beta_{2},\gamma_{2}\right]$$

**Figure 21:**
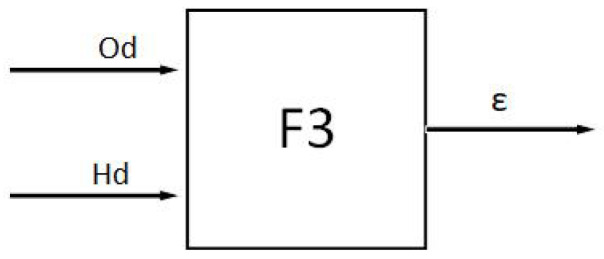
Fusion fuzzy controller.

The fusion fuzzy controller $F_{3}$ is designed, and the structure of the fuzzy controller $F_{3}$ is shown in Figure 21.

When the ferry vehicle is far away from the obstacle and the heading angle is large, we should give priority to the directional principle and take a smaller value of the weight coefficient ε. When the heading angle of the ferry vehicle is small and close to the obstacle, we should give priority to the safety principle and take a larger value of ε. The closer the ferry vehicle is to the obstacle, the more attention that should be paid to safety, and the greater the value of ε. The larger the heading angle of the ferry vehicle is, the higher the emphasis on directionality should be, and the smaller the value of ε should be. When the emphasis on directivity and safety is close, ε should take the appropriate value. The output is defuzzified by the center of gravity method. The specific fusion fuzzy rules are shown in Table 7.

**Figure 22:**
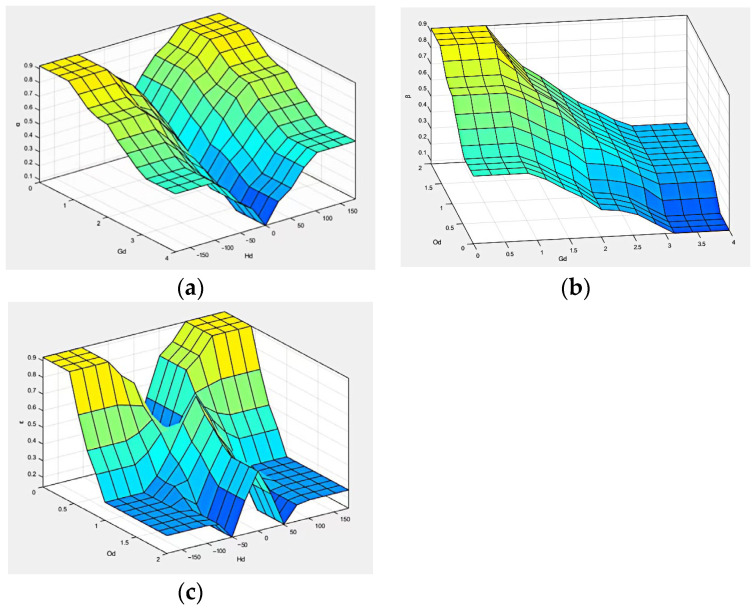
Fuzzy regular three-dimensional surface graph. (**a**) $F_{1}$; (**b**) $F_{2}$; (**c**) $F_{3}$.

Figure 22 shows a three-dimensional surface diagram of fuzzy rules of $F_{1}$, $F_{2}$, and $F_{3}$ fuzzy controllers.

#### 4.2.3. Simulation Experiment of the DWA Algorithm Based on Fuzzy Control

In order to verify that the improved dynamic window algorithm can better adapt to the complex environment than the traditional dynamic window algorithm, the DWA algorithm based on fuzzy control is used to simulate the same environment as in Section 4.1.4. The starting point and target point of the experiment and the parameters of the ferry vehicle are consistent with the traditional dynamic window method. The simulation results in this section are compared with those in Section 4.1.4.

**Figure 23:**
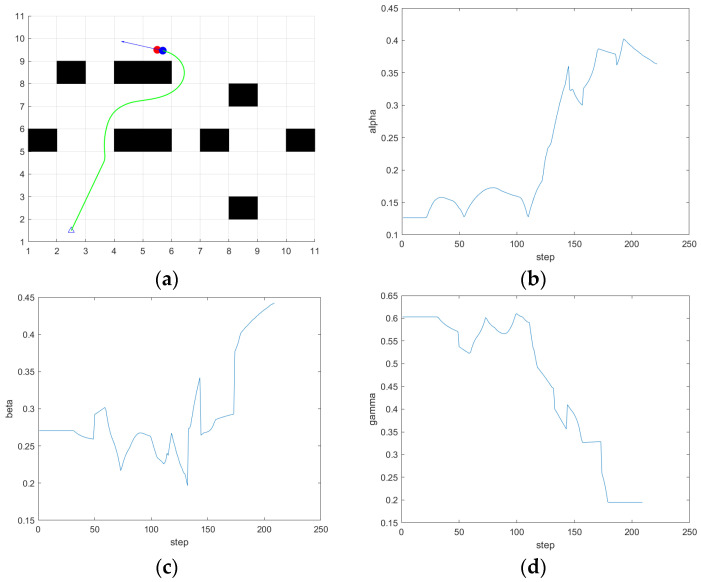
The simulation results of fuzzy DWA in scene 1. (**a**) Path; (**b**) α; (**c**) β; (**d**) γ.

In the first scene, the simulation results are shown in Figure 23, from the path results and the weight coefficient variation curve of the evaluation function, it can be seen that when it is close to 100 steps, the ferry vehicle is close to the obstacle, but there is still a long distance from the target point, so safety is given priority. The α value of the output of the fuzzy controller decreases, and the β value increases. Comply with the fuzzy rules set out in Section 4.2.2. At the same time, as the ferry vehicle is closer to the target point and the obstacles around the target point, the smaller the speed weight γ is, the greater the values of α and β are. Compared with the path simulated by the traditional DWA in scene 1 in Figure 13, the ferry vehicle can dynamically adjust the avoidance weight according to the actual distance from the obstacle on the basis of ensuring the direction so that the ferry vehicle can smoothly bypass the obstacle in front of the target point and reach the target point, so as to avoid a situation where the traditional DWA crosses the obstacle in order to reach the target point faster.

**Figure 24:**
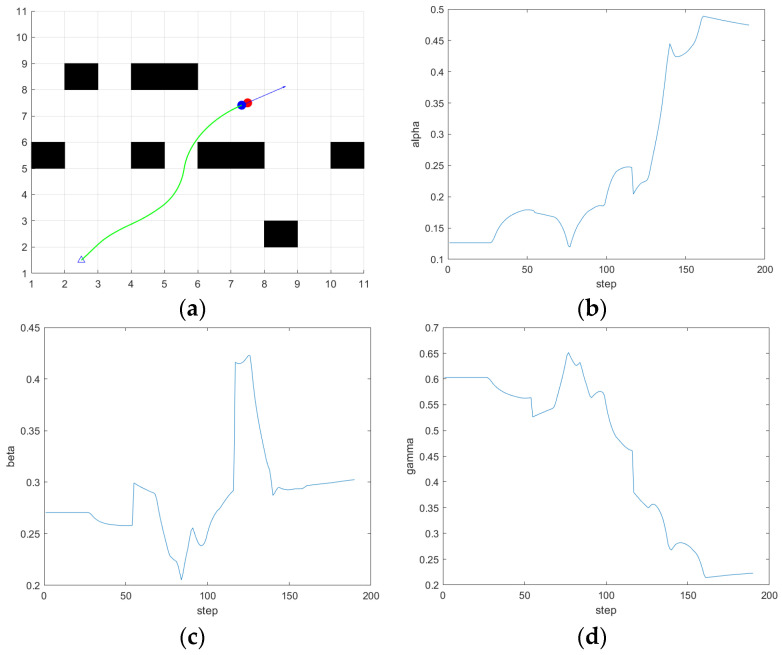
The simulation results of fuzzy DWA in scene 2. (**a**) Path; (**b**) α; (**c**) β; (**d**) γ.

The simulation is carried out in scene 2, the simulation results are shown in Figure 24, and the simulation results show that when there are both obstacles and drivable channels in the direction of the current position of the ferry vehicle facing the target point, that is, the position in the figure is close to 80 steps, the ferry vehicle is close to the obstacle, there is still a moderate distance from the target point, the values of α and β output by the fuzzy controller decrease, and the ferry vehicle chooses the path between the two obstacles. Finally, reach the target point. Avoid the situation where the traditional DWA gives up the optimal path in order to avoid obstacles. The distance and quality of the planned path are effectively optimized.

**Figure 25:**
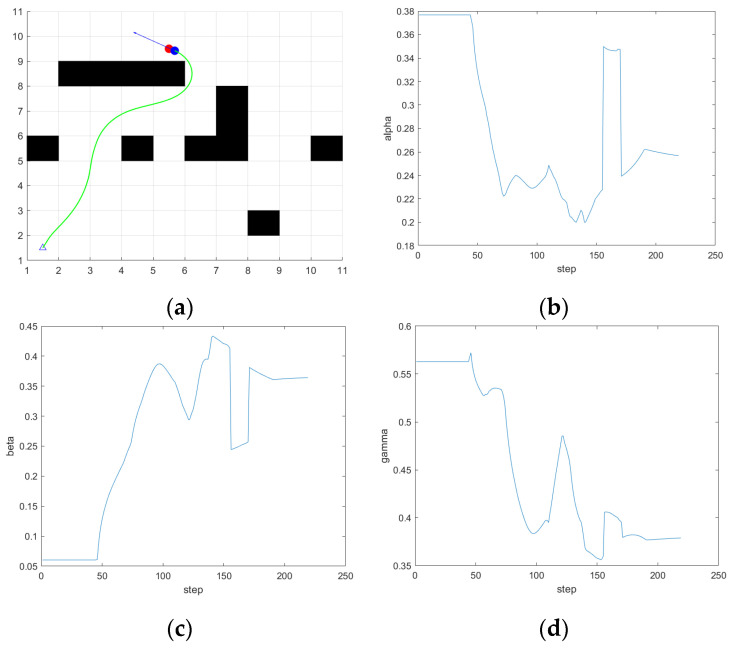
The simulation results of fuzzy DWA in scene 3. (**a**) Path; (**b**) α; (**c**) β; (**d**) γ.

The simulation is carried out in scene 3, the simulation results are shown in Figure 25, and it can be seen from the simulation results that when the ferry vehicle is getting closer and closer to the target point, the speed weight γ of the fuzzy controller will be greatly reduced, the speed of the ferry vehicle will slow down, and the relative heading angle weight will become a moderate value. The weight of avoiding obstacles is increased so that the ferry vehicle can reach the target point smoothly and safely. It will not happen that the ferry bus misses the target point because of the constant speed weight of the traditional DWA.

Through the simulation experiment of the DWA based on fuzzy control, it is obvious that, compared with the traditional DWA, the improved algorithm solves various problems caused by the constant weight coefficient of the evaluation function or the excessive weight coefficient of a certain weight. It solves the problem that the traditional dynamic window method cannot bypass the obstacles or pass through the narrow channel, and thus misses the target point. At the same time, it effectively improves the quality of the planning path.

## 5. A Fusion Algorithm Based on the Improved A* Algorithm and Fuzzy Control DWA 

### 5.1. Algorithm Fusion Principle

**Figure 26:**
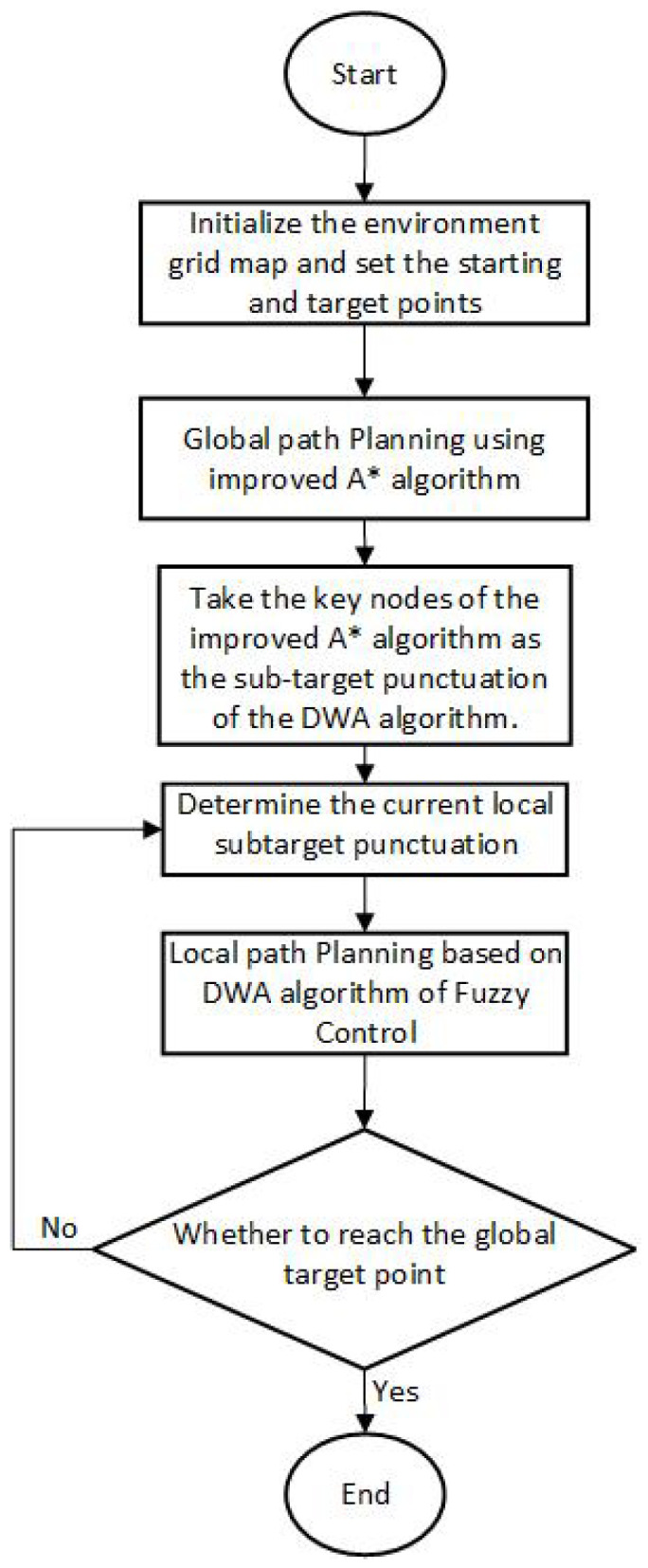
Algorithm fusion flow chart.

The improved A* algorithm can plan the global optimal path in the static scene where the obstacles are known, but it cannot avoid the unknown obstacles in the static scene. The improved DWA can avoid temporary unknown obstacles in real time, but in a large-scale environment with a large number of obstacles, due to the lack of guidance from the intermediate point, it is easy to cause the planning results to fall into local optimization. It is not difficult to find that the integration of global and local planning algorithms can make up for each other’s shortcomings. The key nodes of the path planned by the improved A* algorithm are used as sub-target punctuation points in the local planning of the improved DWA to guide the local path planning. The combination of the two can not only retain global optimality but also avoid unknown obstacles [28]. The specific flow chart of the algorithm fusion is shown in Figure 26.

The specific implementation steps of algorithm fusion are as follows:

(1) The ferry vehicle uses sensors to obtain information about the surrounding environmental obstacles and converts it into environmental raster maps that can be used for path planning.
(2) The improved A* algorithm is used to plan the optimal global path and smooth the path.
(3) Take a key node at the same distance on the planned global path, record the coordinates of all the key nodes, and take them as the sub-target punctuation of the local planning.
(4) According to the acquired local sub-target punctuation, the fuzzy control DWA is used to plan the local path, the surrounding environment information is sensed in real time by the sensors on the ferry vehicle, and the grid map is updated in real time when there are unknown obstacles. Thus, a local path that avoids obstacles can be planned. When the obstacle is bypassed, the ferry vehicle will first return to the global path to continue. If the current local sub-target punctuation is reached, then switch to the next local sub-target punctuation.
(5) Determine whether the currently arriving local sub-target point is a global target point; if not, jump to step 4. Continue the cycle until the ferry reaches the global target point and the algorithm is over.

### 5.2. Simulation Experiment and Result Analysis of the Fusion Algorithm

**Figure 27:**
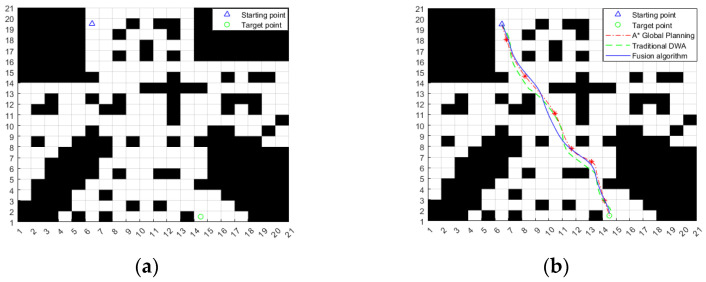
Simulation results of the fusion algorithm in a known static environment. (**a**) Environmental map; (**b**) Simulation results of fusion algorithm.

In order to verify the effectiveness of the fusion algorithm designed in this paper in different environments, considering that the obstacles encountered by ferry vehicles in the process of path planning can be divided into two categories, one is the known static obstacles in the planning environment. Second, there are unknown static obstacles and dynamic obstacles on the basis of the known environment [29]. According to the above situation, two groups of simulation experiments are carried out on the algorithm proposed in this paper. The parameters of the ferry vehicle in the experiment are consistent with the traditional dynamic window method. The two sets of simulations of the path planning of the fusion algorithm use the environment map shown in Figure 27a.

In the first group of simulation environments, there are known static obstacles. The starting point and target point of the ferry vehicle are shown in Figure 27a, and the simulation results are shown in Figure 27b. It can be seen from the figure that the fusion algorithm uses the key nodes of the global path planned by the improved A* algorithm as the local sub-target points of the improved dynamic window method, guides the improved dynamic window method along the global path, successfully bypasses the obstacles, and finally reaches the target point; the path is relatively smooth. However, the paths planned by the traditional DWA and the traditional A* algorithm are too close to or even colliding with the obstacles. It can be seen that it is very necessary to use the fusion algorithm designed in this paper for path planning.

**Figure 28:**
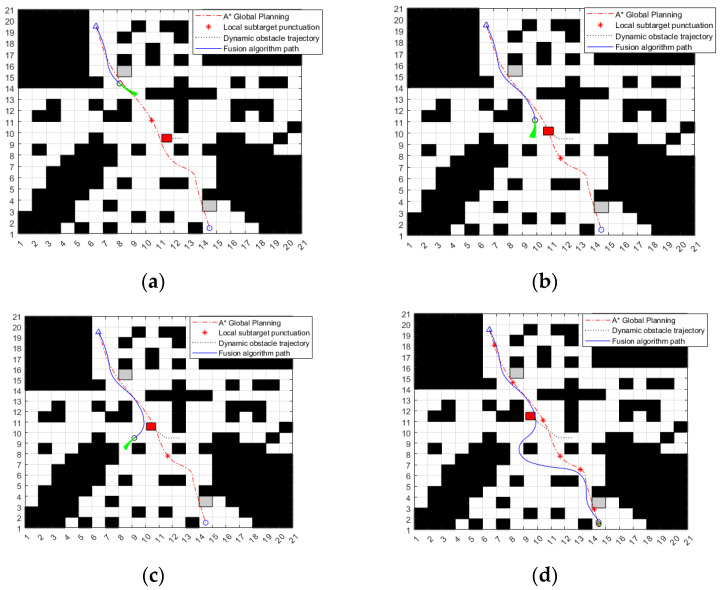
Simulation results of the fusion algorithm in an environment with unknown dynamic and static obstacles. (**a**) Bypass the first static obstacle. (**b**) Encounter dynamic obstacles. (**c**) Bypass dynamic obstacles. (**d**) Bypass the second static obstacle and reach the target point.

The second group of simulation experimental environments simulates that the ferry vehicle plans the global optimal path under the known static obstacle environment, and there are unknown static and dynamic obstacles on the way along the global path. As shown in Figure 28a, the coordinates of the two unknown static obstacles are (8.5, 15.5) and (14.5, 3.5), respectively, represented by a gray grid. The dynamic obstacle moves from (12.5) to (9.5) at the speed of 0.05 m/s, which is represented by a red grid, and the trajectory of the dynamic obstacle is represented by a black dotted line. The prediction trajectory of the fusion algorithm is represented by a green curve. As can be seen from Figure 28, at (a) moment, the ferry vehicle increases the corner to bypass the first static obstacle; at (b) moment, the ferry vehicle adjusts its driving direction after sensing the dynamic obstacle; at (c) moment, the ferry vehicle successfully bypasses the dynamic obstacle and drives to a new route; and at (d) moment, the ferry vehicle bypasses the second static obstacle and reaches the finish line smoothly.

**Figure 29:**
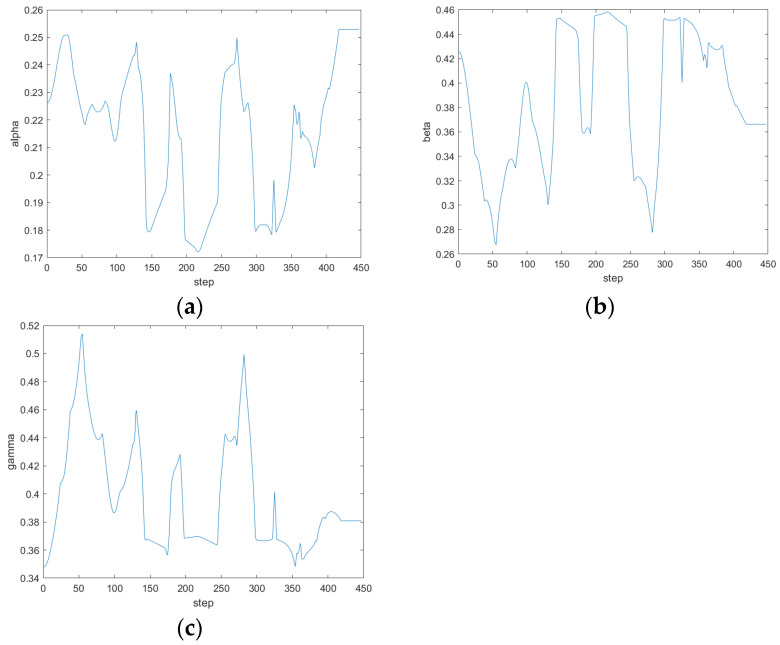
Change of weight coefficient of the fusion algorithm in the environment with unknown dynamic and static obstacles. (**a**) α; (**b**) β; (**c**) γ.

**Figure 30:**
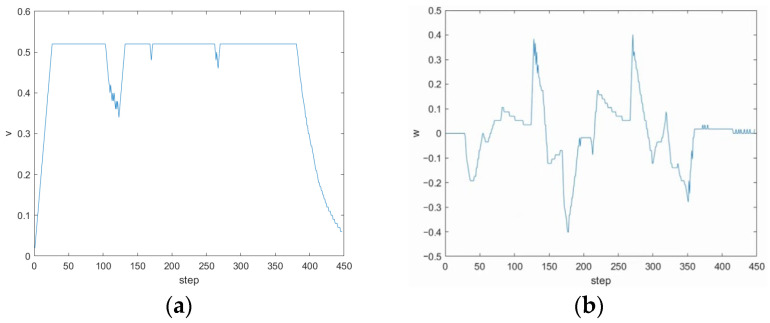
Variation in linear velocity and angular velocity of ferry vehicles. (**a**) Linear velocity variation curve; (**b**) angular velocity variation curve.

During the operation of the fusion algorithm, the changes in the three weight coefficients output by the fuzzy controller are shown in Figure 29, and the linear velocity and angular velocity variation curves of the ferry vehicle are shown in Figure 30. Every 0.1 of linear velocity represents 0.4 m/s, and every 0.1 of angular velocity represents 6 deg/s. When the program iteration starts with 50 steps, the ferry vehicle encounters unknown static obstacles. At this time, the speed weight coefficient γ decreases, the obstacle distance weight coefficient β increases, the heading angle weight coefficient α basically remains stable, and the ferry vehicle tends to slow down and avoid obstacles. The corresponding ferry vehicle maintains the frontline speed to avoid obstacles smoothly, and the angular velocity increases. When the procedure iterates to 120 steps, the ferry vehicle meets the dynamic obstacle at first, α and γ are in the large value, and β is in the middle value; that is, the ferry vehicle tends to drive faster towards the target point, and the weight of obstacle avoidance is relatively low. But as the dynamic obstacle approaches the ferry vehicle, the values α and γ begin to decrease greatly, and the value β increases greatly. The primary task of the ferry vehicle is to avoid the dynamic obstacle; the speed and heading angle weight decrease, and the corresponding speed of the ferry vehicle decreases. At the same time, the angular velocity increases when turning away from the target and avoiding the obstacle. In addition, in the process of avoiding dynamic obstacles, the three weight coefficients change violently according to the relative relationship between ferry vehicles and dynamic obstacles. When approaching the target point, the heading angle weight coefficient becomes larger, the speed weight coefficient decreases, the obstacle weight coefficient is moderate, and the three coefficients are maintained in a relatively stable range. The ferry vehicle successfully avoids the second unknown static obstacle and reaches the target point.

**Figure 31:**
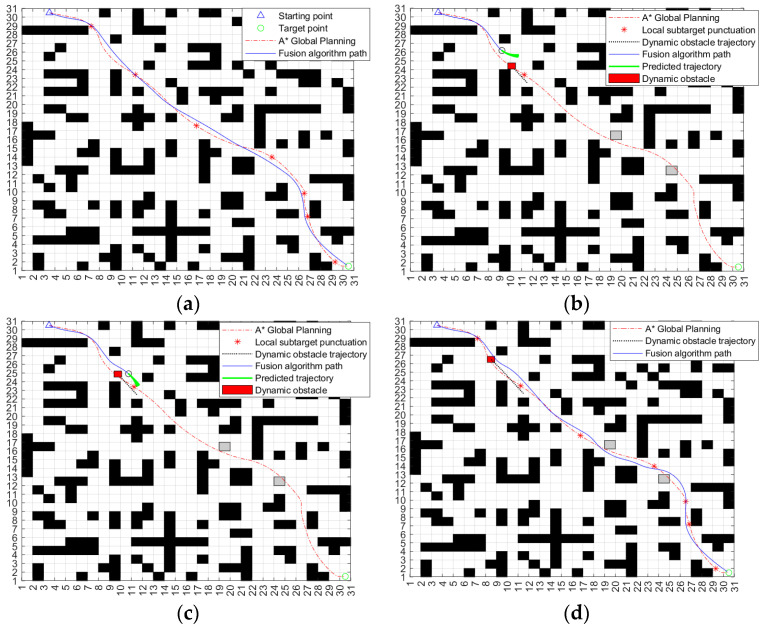
Simulation experiment results of a 30 × 30 environment map. (**a**) Environment without unknown obstacles. (**b**) Encounter dynamic obstacles and turn. (**c**) Successfully circumvent dynamic obstacles. (**d**) Move the static obstacle around and reach the target point.

The above two sets of simulation experiments verify the effectiveness of the fusion algorithm when there are no unknown obstacles and unknown dynamic and static obstacles in the same map. In order to further verify the adaptability of the algorithm under different maps [30], a more complex 30 × 30 environment map is selected for the simulation experiment. The environmental map is also divided into two cases: no unknown obstacles and unknown dynamic and static obstacles. The experimental results are shown in Figure 31.

**Figure 32:**
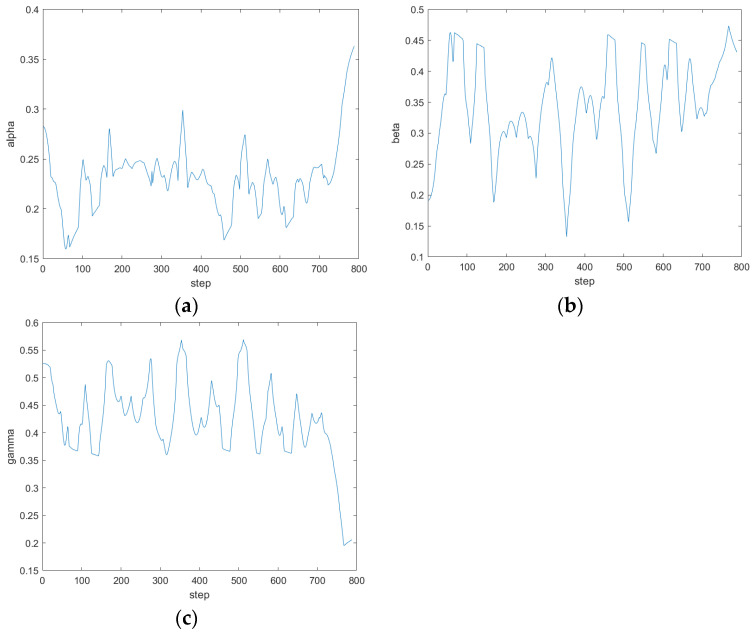
The change in output weight coefficient in the presence of unknown dynamic and static obstacles. (**a**) α; (**b**) β; (**c**) γ.

In the environment without unknown obstacles in Figure 31a, the fusion algorithm can plan a complete path according to the key nodes in the global path of the improved A* algorithm. Compared with the global path planned by the A* algorithm, the distance between the path and obstacles planned by the fusion algorithm is safer, the path smoothness is higher, and it is more suitable for ferry vehicle tracking. Figure 31b–d adds unknown dynamic and static obstacles to the environment of Figure 31a. As can be seen from the map, the path planned by the improved A* algorithm cannot avoid unknown obstacles. In addition, through experiments, the traditional DWA cannot successfully plan a complete path from the starting point to the target point on such a complex map. Through Figure 31b,c, it can be seen that the ferry vehicle can adjust its driving route in time and bypass the dynamic obstacles when it encounters them. At the same time, it can quickly return to the original path according to the key points of the improved A* algorithm after the danger is lifted. As can be seen from Figure 31d, the path planned by the fusion algorithm can successfully bypass two unknown static obstacles and reach the target point. The whole path is safe and reliable. The changes in the three weight coefficients of the fuzzy controller output during the operation of the fusion algorithm are shown in Figure 32.

**Figure 33:**
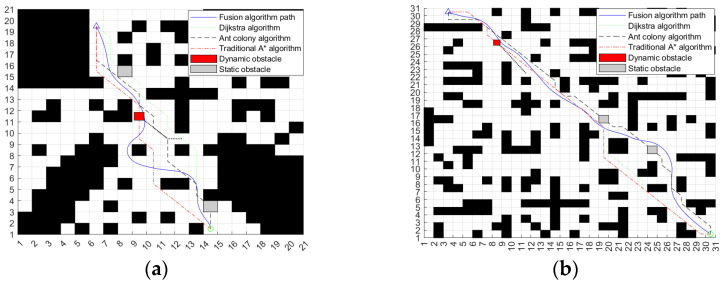
Simulation and comparison results of different algorithms. (**a**) 20 × 20 environment map; (**b**) 30 × 30 environment map.

In order to verify whether the path planned by the fusion algorithm has sufficient advantages, in the environment of the above two different environmental maps and dynamic and static obstacles, the simulation experiments are carried out by using the Dijkstra algorithm, ant colony algorithm, and traditional A* algorithm, respectively, and the obtained path is shown in Figure 33.

The performance comparison of the four algorithms is shown in Table 8. It can be seen that in addition to the fusion algorithm, the remaining three traditional algorithms plan the risk path of collision in the face of unknown dynamic and static obstacles, and only the fusion algorithm can plan a safe collision-free path. And in terms of the number of path turns, path length, and algorithm time, compared with other traditional algorithms, the path length planned by the fusion algorithm is shorter, the number of turns is lower, and the path is smoother. It is more suitable for ferry vehicles to follow. Moreover, the algorithm takes less time and has higher efficiency, which fully proves that the improved fusion algorithm is effective.

Through the above experiments on the distribution of different obstacles under the same map, it is proved that the improved fusion algorithm is effective in the face of unknown dynamic and static obstacles, and by improving the size and complexity of the map, the adaptability of the fusion algorithm in different scenes is verified; compared with several traditional algorithms, the performance of the fusion algorithm is superior to the common traditional algorithms.

According to the above experiments, the fusion algorithm solves problems that traditional algorithms cannot, such as the A* algorithm’s inability to avoid unknown obstacles and the DWA easily falling into local optimization in a large area environment, and the effectiveness and adaptability of the algorithm are verified.

## 6. Conclusions

This paper presents a path planning algorithm based on the fusion of the improved A* and DWA algorithms for driverless ferry vehicles. In order to solve the traditional A* algorithm, the search direction is not high, the search time is long, the path is not smooth, and it is only suitable for static maps and other problems.

(1) In the aspect of global planning, the vector angle cosine value is introduced to improve the heuristic function on the basis of the traditional A* algorithm, which reduces the inspection of useless nodes and makes the path planning more directional. In the traditional A* algorithm, the search neighborhood of 3 × 3 is extended to the search neighborhood of 9 × 9, which shortens the search time, improves the search efficiency, and optimizes the path length. Then the path is smoothed based on a quasi-uniform cubic B-spline curve, which makes the planned path more suitable for ferry vehicle tracking. The effectiveness of the improved algorithm is verified by simulation experiments.
(2) In the aspect of local planning, the problems caused by the traditional DWA algorithm using constant weight coefficients in different environments are analyzed through simulation experiments. Fuzzy control theory is introduced to improve the traditional DWA algorithm. Based on the principles of directionality and safety, three fuzzy controllers are designed to integrate the two principles to realize the dynamic adjustment of the three weight coefficients of the trajectory evaluation function in different environments. Then the simulation results show that the improved DWA algorithm can solve the problems of the traditional DWA algorithm, such as being unable to bypass obstacles, unable to pass through narrow channels, and missing target points. It effectively improves the quality of the planning path.
(3) In the aspect of algorithm fusion, in order to solve the problem that the global planning algorithm cannot avoid unknown obstacles and the local planning algorithm is easy to fall into local optimization because of the lack of intermediate point guidance in large-scale environments, a fusion algorithm based on an improved A* algorithm and a fuzzy control DWA algorithm is proposed. The simulation results show that the path planned by the fusion algorithm not only fits the global static optimal path but can also effectively avoid the unknown dynamic and static obstacles in the path. At the same time, it still has a good effect on obstacle avoidance planning in the more complex environmental map, which fully verifies the effectiveness and adaptability of the fusion algorithm in ferry vehicle path planning.

Overall, this paper provides a feasible and effective solution to improve the path planning performance of driverless ferry vehicles, which has potential significance for the application of driverless ferry vehicles.

In view of the fact that the improved DWA algorithm based on fuzzy control depends on subjective experience in formulating fuzzy rules, it is sometimes necessary to adjust the fuzzy rules according to the empirical changes in the actual verification process. In the next research, we can combine the deep learning algorithm to deeply optimize the fuzzy rules of the weight coefficient of the DWA algorithm to realize the adaptive adjustment of the fuzzy rules, so as to further increase the adaptability of the algorithm. At the same time, the study of this paper only aims at a single ferry vehicle, which has limitations, and more in-depth research can be carried out from the perspective of multi-ferry vehicle formation path planning in the future.

## Figures and Tables

**Table 1:** Planning path result.

Arithmetic	Number of Inflection Points	Path Length/m	Planning Time/s
Traditional A*	9	21.98	0.91
Improve A*	7	21.77	0.87

**Table 2:** Planning path result.

Search Neighborhood	Included Node	Path Length/m	Planning Time/s
3 × 3	31	21.77	0.87
5 × 5	29	21.31	0.74

**Table 3:** Map 1 planning path results.

Search Neighborhood	Adjacent Node	Searchable Direction	Path Length/m	Planning Time/s
3 × 3	8	8	20.42	0.61
5 × 5	24	16	20.31	0.46
7 × 7	48	32	19.77	0.43
9 × 9	80	56	19.60	0.40

**Table 4:** Map 2 planning path results.

Search Neighborhood	Adjacent Node	Searchable Direction	Path Length/m	Planning Time/s
3 × 3	8	8	21.77	0.87
5 × 5	24	16	21.31	0.74
7 × 7	48	32	20.61	0.69
9 × 9	80	56	20.36	0.60

**Table 5:** Directional fuzzy rule table.

Input Gd	Input Hd	$\mathrm{Output}\;\alpha_{1}$	$\mathrm{Output}\;\gamma_{1}$
N	XS or XB	XL	S
N	S or B	L	S
N	M	M	S
M	XS or XB	L	M
M	S or B	M	M
M	M	S	M
F	XS or XB	M	L
F	S or B	S	L
F	M	XS	L

**Table 6:** Security fuzzy rule table.

Input Od	Input Gd	$\mathrm{Output}\;\beta_{2}$	$\mathrm{Output}\;\gamma_{2}$
N	N	M	S
N	M	L	M
N	F	XL	M
M	N	S	S
M	M	M	M
M	F	M	L
F	N	XS	M
F	M	S	L
F	F	S	L

**Table 7:** Fusion fuzzy rule table.

Input Od	Input Hd	ε
N	XS or XB	XL
N	S or B	L
N	M	L
M	XS or XB	S
M	S or B	M
M	M	L
F	XS or XB	S
F	S or B	XS
F	M	M
N	XS or XB	XL
N	S or B	L
N	M	L
M	XS or XB	S
M	S or B	M
M	M	L
F	XS or XB	S
F	S or B	XS
F	M	M

**Table 8:** Performance parameters of four algorithms.

Map Size	Algorithm Category	Number of Turning Points	Path Length/m	Is There a Risk Path	The Algorithm Take Time/s
20 × 20	Fusion algorithm	5	23.97	no	12.76
	Dijkstra algorithm	9	21.43	yes	14.11
	Ant colony algorithm	10	22.87	yes	19.35
	Traditional A* algorithm	6	21.36	yes	13.78
30 × 30	Fusion algorithm	7	40.83	no	22.55
	Dijkstra algorithm	10	41.32	yes	26.53
	Ant colony algorithm	15	42.27	yes	33.43
	Traditional A* algorithm	7	41.03	yes	24.83

## Data Availability

The data used to support the findings of this study are available from the corresponding author upon request.

## References

[1] Singh S., Saini B.S. (2021). Autonomous cars: Recent developments, challenges, and possible solutions. IOP Conf. Ser. Mater. Sci. Eng..

[2] Agriesti S., Brevi F., Gandini P., Marchionni G., Parmar R., Ponti M., Studer L. (2020). Impact of Driverless Vehicles on Urban Environment and Future Mobility. Transp. Res. Procedia.

[3] Yimer T.H., Wen C., Yu X., Jiang C.J. (2020). A study of the minimum safe distance between human driven and driverless cars using safe distance model. arXiv.

[4] Sanchez-Ibanez J.R., Perez-Del-Pulgar C.J., Garcia-Cerezo A. (2021). Path Planning for Autonomous Mobile Robots: A Review. Sensors.

[5] Luo M., Hou X.R., Yang J. (2020). Surface Optimal Path Planning Using an Extended Dijkstra Algorithm. IEEE Access.

[6] Sudhakara P., Ganapathy V. (2016). Trajectory planning of a mobile robot using enhanced A-star algorithm. Indian J. Sci. Technol..

[7] Mashayekhi R., Idris M.Y.I., Anisi M.H., Ahmedy I., Ali I. (2020). Informed RRT*-Connect: An Asymptotically Optimal Single-Query Path Planning Method. IEEE Access.

[8] Miao C.W., Chen G.Z., Yan C.L., Wu Y.Y. (2021). Path planning optimization of indoor mobile robot based on adaptive ant colony algorithm. Comput. Ind. Eng..

[9] Keyu L., Yonggen L., Yanchi Z. Dynamic obstacle avoidance path planning of UAV Based on improved APF. Proceedings of the 2020 5th International Conference on Communication, Image and Signal Processing (CCISP).

[10] Wu J., Ma X., Peng T., Wang H. (2021). An Improved Timed Elastic Band (TEB) Algorithm of Autonomous Ground Vehicle (AGV) in Complex Environment. Sensors.

[11] Lai X., Wu D., Wu D., Li J.H., Yu H. (2023). Enhanced DWA algorithm for local path planning of mobile robot. Ind. Robot.

[12] Tang G., Tang C.Q., Claramunt C., Hu X., Zhou P.P. (2021). Geometric A-Star Algorithm: An Improved A-Star Algorithm for AGV Path Planning in a Port Environment. IEEE Access.

[13] Wu D.H., Wei L.S., Wang G.L., Tian L., Dai G.Z. (2022). APF-IRRT*: An Improved Informed Rapidly-Exploring Random Trees-Star Algorithm by Introducing Artificial Potential Field Method for Mobile Robot Path Planning. Appl. Sci..

[14] Dai J., Li D., Zhao J., Li Y. (2022). Autonomous Navigation of Robots Based on the Improved Informed-RRT* Algorithm and DWA. J. Robot..

[15] Shang E.K., Bin D., Nie Y.M., Qi Z., Liang X., Zhao D.W. (2020). An improved A-Star based path planning algorithm for autonomous land vehicles. Int. J. Adv. Robot Syst..

[16] Jiao C.J., Heitzler M., Hurni L. (2021). A survey of road feature extraction methods from raster maps. Trans. GIS.

[17] Ju C., Luo Q., Yan X. Path planning using an improved a-star algorithm. Proceedings of the 2020 11th International Conference on Prognostics and System Health Management (PHM-2020 Jinan).

[18] Tang X., Zhu Y., Jiang X. (2021). Improved A-star algorithm for robot path planning in static environment. J. Phys. Conf. Ser..

[19] Li L. (2020). Application of Cubic B-spline Curve in Computer-Aided Animation Design. Comput. Aided Des. Appl..

[20] Qin K. General matrix representations for B-splines. Proceedings of the Proceedings Pacific Graphics’ 98. Sixth Pacific Conference on Computer Graphics and Applications (Cat. No. 98EX208).

[21] Zhang F., Li N., Xue T., Zhu Y., Yuan R., Fu Y. An improved dynamic window approach integrated global path planning. Proceedings of the 2019 IEEE International Conference on Robotics and Biomimetics (ROBIO).

[22] Ren X., Cai Z. Kinematics model of unmanned driving vehicle. Proceedings of the 2010 8th World Congress on Intelligent Control and Automation.

[23] Schramm D., Hiller M., Bardini R.J.M. (2014). Vehicle Dynamics: Modeling and Simulation.

[24] Xu Z., Wei Y. (2022). Mobile robot path planning based on fusion of improved A* algorithm and adaptive DWA algorithm. Proc. J. Phys. Conf. Ser..

[25] Kovacic Z., Bogdan S. (2018). Fuzzy Controller Design.

[26] Dubois D., Prade H. (1996). What are fuzzy rules and how to use them. Fuzzy Set Syst..

[27] Iancu I. (2012). A Mamdani type fuzzy logic controller. Fuzzy Logic—Controls, Concepts, Theories and Applications.

[28] Zhu Z., Xie J., Wang Z. Global dynamic path planning based on fusion of a* algorithm and dynamic window approach. Proceedings of the 2019 Chinese Automation Congress (CAC).

[29] Said A., Talj R., Francis C., Shraim H. Local trajectory planning for autonomous vehicle with static and dynamic obstacles avoidance. Proceedings of the 2021 IEEE International Intelligent Transportation Systems Conference (ITSC).

[30] Li D., Shi X., Dai M. An Improved Path Planning Algorithm Based on A* Algorithm. Proceedings of the International Conference on Computer Engineering and Networks.

